# The role of social determinants of health in mental health: An examination of the moderating effects of race, ethnicity, and gender on depression through the all of us research program dataset

**DOI:** 10.1371/journal.pmen.0000015

**Published:** 2024-08-05

**Authors:** Matt Kammer-Kerwick, Kyle Cox, Ishani Purohit, S. Craig Watkins

**Affiliations:** 1 IC^2^ Institute, The University of Texas at Austin, Austin, Texas, United States of America; 2 Moody School of Communications and IC^2^ Institute, The University of Texas at Austin, Austin, Texas, United States of America; King’s College London, UNITED KINGDOM OF GREAT BRITAIN AND NORTHERN IRELAND

## Abstract

We investigate how select identity characteristics moderate the role of several SDoH domains on major depressive disorder (MDD). Our study considers an analytical sample of 86,954 participants from the NIH-funded All of Us (AoU) Research Program in the USA. Our independent variables and moderators come from survey responses and our outcome is an EHR diagnostic code. We include race/ethnicity and gender/sexual identity to moderate the role of food insecurity, discrimination, neighborhood social cohesion, and loneliness in assessing risk for MDD diagnosis. We examine those moderating effects based on connections seen in the literature. Our findings illustrate the complexity of where and how people live their lives can have significant differential impact on MDD. Women (AOR = 1.60, 95% CI = [1.53, 1.68]) and LGBTQIA2+ individuals (AOR = 1.71, 95% CI = [1.60, 1.84]) exhibit a significantly higher likelihood of MDD diagnosis compared to cisgender heterosexual males. Our study also reveals a lower likelihood of MDD diagnosis among Asian/Asian American individuals (AOR = 0.41, 95% CI = [0.35, 0.49]) compared to White individuals. Our results align with previous research indicating that higher levels of food insecurity (AOR = 1.30, 95% CI = [1.17, 1.44]) and loneliness (AOR = 6.89, 95% CI = [6.04, 7.87]) are strongly associated with an increased likelihood of MDD. However, we also find that social cohesion (AOR = 0.92, 95% CI = [0.81, 1.05]) does not emerge as a significant predictor, contradicting some literature emphasizing the protective role of neighborhood cohesion. Similarly, our finding that transience (AOR = 0.95, 95% CI = [0.92, 0.98]) reduces the likelihood of MDD diagnosis contradicts conventional wisdom and warrants further exploration. Our study provides a reminder of the substantial challenges for research focused on marginalized community segments and that deliberate sampling plans are needed to examine those most marginalized and underserved.

## Introduction

In 2008, the World Health Organization (WHO) codified notions of health equity and “the conditions in which people are born, grow, live, work, and age” as the social determinants of health (SDoH) [1]. In the nearly 15 years, significant scholarship and policy efforts have been devoted around the globe to improve understanding and gauge best practices for achieving improvement in health equity based on therapeutic area, patient population, and various typologies for the determinant domains included. The WHO currently lists the following as “examples of the social determinants of health, which can influence health equity in positive and negative ways: income and social protection; education; unemployment and job insecurity; working life conditions; food insecurity; housing, basic amenities, and the environment; early childhood development; social inclusion and non-discrimination; structural conflict; and access to affordable health services of decent quality [2].” U.S. Department of Health and Human Services’ Healthy People 2030 program [3] states that SDoH “can be grouped in to 5 domains: economic stability, education access and quality, healthcare access and quality, neighborhood and built environment, and social and community context.” These two perspectives serve to illustrate that there are different taxonomies for SDoH. For the interested reader, in their study of diabetes, Hill-Briggs and colleagues (2021) compare and contrast the SDoH schema by the WHO, Healthy People 2020, County Health Rankings Model (2014), and Kaiser Family Foundation (2018) [4].

Foundational research by Link and Phelan (1995) highlighted the persistent influence of socioeconomic status as a fundamental cause of health inequalities. These studies suggest that resources such as knowledge, money, power, prestige, and beneficial social connections, which are unevenly distributed across socioeconomic strata, directly impact health outcomes across diverse contexts and diseases [5]. Further expanding on this, Braveman and Gottlieb (2014) discussed the underlying socioeconomic causes of disparities observed in SDoH, proposing that gaps in income and education are primary drivers of health inequities rather than mere manifestations within different SDoH frameworks [6]. This line of inquiry critically examines the traditional characterization of variables as mere socioeconomic or demographic factors, pointing to a deeper causality where structural inequalities fundamentally shape health outcomes. This perspective supports a moderation approach in our research, highlighting the need for policy interventions that not only address the symptoms but also the roots of health disparities.

Further exploration of SDoH has illuminated their significant role in moderating the relative risks of chronic diseases such as cardiovascular disease, diabetes, cancer, and mental health issues [4,7,8]. For example, researchers have examined low socioeconomic status and unsafe neighborhoods are risk factors that make some populations more susceptible to developing chronic illnesses. Understanding and addressing these determinants is crucial for promoting health equity and ensuring that all individuals have the opportunity to lead healthy, fulfilling lives.

In this study, we adopt an intersectional approach to analyze how various demographic characteristics—such as race/ethnicity, gender/sexual identity, income, and age—interact with SDoH to influence the prevalence and severity of major depressive disorder (MDD). Through correlational moderation analysis, we identify specific contexts in which certain SDoH have a more pronounced relationship with MDD. This approach not only allows us to reveal important interactions between SDoH and individual demographic factors but also to acknowledge the limitations of quantitative methods in fully capturing the breadth of intersectional impacts.

Collectively, across this literature, we believe that there is opportunity to further examine the interplay between socioeconomic and demographic factors with various SDoH through the lens of intersectionality [9,10]. We address this opportunity in the current study by addressing when, how, and for whom specific SDoH matter. We examine how population characteristics like race/ethnicity, gender/sexual identity, income, and age influence the connection between SDoH and a specific, highly prevalent mental health condition, major depressive disorder (MDD).

Specifically, the current study probes the effects of SDoH in relation to MDD using data from the All of Us (AoU) Research Program [11]. MDD is one of the most common forms of mental health disorders in the U.S. According to the NIMH, 21 million U.S. adults, or 8.4% of all adults, had at least one major depressive episode in 2020 [12]. The distribution of MDD varies noticeably among adults in the U.S. For example, from the same report, women (10.5%) have higher levels of MDD compared to men (6.2%). Similarly, young adults ages 18–25 suffer higher rates of MDD than their older counterparts aged 50 and older (5.4%). There are also racial and ethnic differences in the prevalence of MDD among U.S. adults. In terms of race and ethnicity, persons who report two or more racial/ethnic identities have the highest (15.9%) prevalence of MDD compared to white individuals (9.5%), Latinx individuals (7.0%), Black individuals (6.0%), and Asian individuals (4.2%) [12].

### The current study

While there’s a consensus that Social Determinants of Health (SDoH) influence health outcomes, we need more empirical studies to delve into the complexities of these interactions. Not all SDoH are equal in prevalence, distribution, or impact. It’s highly likely that their effects vary depending on population characteristics. For instance, women with children may face different vulnerabilities to certain SDoH compared to women without children. We must strive to understand with precision when, how, and for whom specific SDoH matter. Alegria et al. (2018) emphasize the need to investigate how SDoH affect different populations [13].

These considerations led our team to address the following research questions in our analysis of the All of Us Research Program dataset:

RQ1. To what degree, if at all, do demographic characteristics predict risk of depression?
RQ2. To what degree, if at all, do SDoH factors predict risk of depression?
RQ3. How do race/ethnicity and gender identity/sexual orientation moderate the role of social determinants of health on risk of depression?

Specifically, to address health equity, in RQ3, we focus on the moderating effects of race/ethnicity and gender identity/sexual orientation while controlling for other demographic factors. Based on our review of the literature, and the relationships with depression found therein, we examine those moderating effects on food insecurity, discrimination, neighborhood social cohesion, and loneliness. To be clear, while literature provides a justification for our choices, other SDoH are connected to depression, a point made in our limitations section.

## Background

To set the stage for the current study, we review depression, as exacerbated by COVID and as an enduring societal and health challenge; the complex connections between various SDoH and depression; and the resources available in the All of Us Research Program dataset [11] that we utilize to probe SDoH in relation to depression.

### Depression

The COVID-19 pandemic has significantly affected the mental well-being of people globally, leading to a surge in Major Depressive Disorder (MDD). In the United States, the rates of MDD have surged due to pandemic-induced changes such as increased social isolation and economic stress [14,15]. The severity of COVID-19’s impact on health appears linked to gender, with some gender-related moderating effects [16,17]. Additionally, the pandemic exposed the health disparities stemming from systemic inequalities [18]. The National Center for Health Statistics (NCHS) and the U.S. Census Bureau conducted the Household Pulse Survey, revealing that 23.6% of respondents reported MDD symptoms in December 2022 [12]. This survey also highlighted that marginalized groups experienced a sharper decline in mental health during the pandemic, with higher rates of MDD symptoms among women, LGBTQ+ individuals, and certain racial/ethnic groups.

Beyond the influence of COVID-19, these findings suggest that MDD is intertwined with broader societal and population factors. MDD has severe consequences, including an increased mortality risk of 60–80% [19]. This risk extends beyond suicide and encompasses cardiovascular death and stroke [20]. Depression also carries significant economic implications, affecting income and employment due to decreased productivity and increased absenteeism, resulting in an annual economic loss of $36.6 billion in the U.S. [21,22].

### SDoH and depression

Various studies have established a link between SDoH and mental illness. Gnanapragasam et al. (2021) highlights crucial social determinants of mental health, including adverse education, employment, poverty, food insecurity, housing conditions, and discrimination [23]. Similarly, Compton and colleagues (2015) identify core SDoH of mental health, such as racial discrimination, early life experiences, education, employment, poverty, housing quality, and access to healthcare [24]. Jeste and Pender (2022) add factors like homelessness, social isolation, positive childhood experiences, and community-level resilience as contributors to the outcomes for individuals with serious mental illnesses and substance use disorders [25]. African American students have been shown to be more likely to experience food insecurity compared to their Caucasian peers. Moreover, students facing severe food insecurity had a 4.52-times higher likelihood of developing depression compared to those with more food security [26]. Neighborhood cohesion has been shown to have a more significant protective effect for heterosexual groups, particularly against moderate psychological distress, in comparison to their non-heterosexual counterparts [27]. Multifactorial discrimination was a significant risk factor for high depression scores, particularly in terms of chronic strain and the total number of stressful life events. Notably, women and Latino/Hispanic individuals were more likely to have high depression scores, highlighting the involvement of social identities and their influence on mental health [28]. Significant research has examined the strong connection between loneliness and depression examining the role in both young adults [29] and the elderly [30]. Indeed, loneliness and depression are often comorbid and connected with other factors like education, wealth, and status [31]. Box A in S1 Text contains additional details on the connections between food insecurity, neighborhood social cohesion, discrimination, and loneliness on depression that are the focus of the current study. We limit the current study to these SDoH in part because of relationships described here but also due to parsimony. As such, we recognize that other SDoH should be similarly examined, a subject we return to in our limitations.

### Examination of SDoH and depression through the all of us research program dataset

Researchers have used the All of Us Research Program data to study a broad range of topics, but few studies have focused on the use of this resource to examine SDoH and depression. Chan and colleagues looked at depression in the context of ocular surgery without utilizing the AoU SDoH survey [32]. Cook and colleagues examined depression in the context of the COVID pandemic, utilizing select measures from the AoU SDoH survey (discrimination, resilience, social support, and loneliness) [33]. The most specific of these studies, by Barr and colleagues, assessed the prevalence of various psychiatric diagnoses in the All of Us dataset compared to other published estimates [34]. There are multiple numbers of mental health diagnosis in the All of Us dataset including, for example, MDD, bipolar, generalized anxiety disorder, and PTSD. Barr and colleagues [34] examine disparities across sociodemographic factors that were ultimately found to be similar to disparities from nationally representative samples. Non-Hispanic White, female at birth, women, and LGBTQ individuals in the sample were at increased risk of most disorders. However, Black, multiracial, and other non-White participants were particularly susceptible to schizophrenia which may be attributed to racism and biases in diagnoses [34]. Indeed, other studies have also found such racial disparities to be from bias in clinical trials and access to care [35]. Having a college degree and an annual household income more than $100,000 served as protective factors that decreased risk for each disorder. Their paper concludes with positive endorsement of the robustness of the AoU data relative to the general population.

## Method

We developed a computational workflow in the AoU workbench environment to query tables to create an analytical dataset [36]. In this section we describe the analytical sample used for the study and document how we operationalized features for our analysis. Our Python and R computational methods are documented and available from the lead author on the AoU platform. AoU participant data cannot be downloaded from the AoU platform, but our dataset and models can be recreated by sharing our workspace.

### Ethics statement

The current study uses nonidentifiable human subjects’ data provided by the NIH All of Us Research Program. These data and the computational notebooks we used are available through that public resource. The AoU Research Program has very formal and transparent privacy policies, see https://allofus.nih.gov/protecting-data-and-privacy for details. All four authors are up to date on their human subjects training as required by our institution, The University of Texas at Austin. The first and second authors accessed and analyzed AoU data for this study. Both completed all the additional training required by NIH to access AoU data and create and utilize the AoU computational notebook spaces. Our analyses and reporting complied with all the AoU reporting requirements. We have complied with requirements to not report summary statistics for small cell sizes. The analysis presented in the current study used the AoU data that was available in June of 2023.

### All of us research program

The All of Us Research Program explicitly addresses the need for greater diversity in medical research [11,37]. The All of Us Research Program has recruited adult participants from partner sites in all 50 states in the US. Participants enroll through the program’s website and can opt in to sharing EHR data and completing several survey modules. Mapes et al. (2020) review the scope of the diversity design in the program and describe efforts made to ensure that participants reflect the diversity of the US [38]. The All of Us Research Program added its SDoH questionnaire in its third year [39]. As such, its design was additive to the surveys implemented earlier that include questions germane to SDoH. Additionally, the sample base of participants who have completed the SDoH survey is a subset of the total sample. The All of Us SDoH survey consists of 81 questions which are drawn from several different instruments that are listed in the Box B and Table A in S1 Text. Additional details about these instruments are available at the All of Us website.

Our analytical sample included 86,954 participants. Table 1 describes our sample in terms of the prevalence of an MDD diagnosis, gender and sexual identity, race and ethnic, age, income, college degree, and home ownership. Table 1 also includes the prevalence of an MDD diagnosis for all of the demographic variables in the table. The overall prevalence of an MDD diagnosis was 14.1%. While diversity is evident across the sample, compared to the US population, participation does skew toward female, white, older, wealthier, college educated, and towards home ownership. Further, rates of MDD diagnosis vary by demographic segment. Descriptively, cisgender heterosexual (CH) females and those with a minoritized gender or sexual identity (GSM) have higher rates of MDD diagnosis than CH males. Asians have the lowest rate of MDD diagnosis compared to other racial and ethnic identities. MDD diagnosis rates are the highest among those in their forties and fifties, increasing from young adulthood and then declining among those 65 or older. Increasing household income (HHI) corresponds to lower rates of MDD diagnosis. We examine these variables and these MDD-related patterns further in the models presented below.

**Table 1:** AoU participant composition and prevalence of MDD diagnosis.

		Sample Distribution	Prevalence of MDD Rx
Variable	N	N = 86,954 n (%) (95% CI)^*1*,*2*^	N = 12,288 n / N (%) (95% CI)^*3*,*4*^
**MDD**	86,954		
Not Depressed		74,666 (85.9%) (85.6%, 86.1%)	
Depressed		12,288 (14.1%) (13.9%, 14.4%)	
**Gender and Sexual Identity**	86,954		
CH male		27,575 (31.7%) (31.4%, 32.0%)	2,779 / 27,575 (10.1%) (9.73%, 10.4%)
CH female		50,063 (57.6%) (57.2%, 57.9%)	7,883 / 50,063 (15.7%) (15.4%, 16.1%)
GSM		9,316 (10.7%) (10.5%, 10.9%)	1,626 / 9,316 (17.5%) (16.7%, 18.2%)
**Race/Ethnicity**	86,954		
White		69,338 (79.7%) (79.5%, 80.0%)	9,984 / 69,338 (14.4%) (14.1%, 14.7%)
Asian		2,332 (2.7%) (2.58%, 2.79%)	136 / 2,332 (5.8%) (4.93%, 6.88%)
Black		5,978 (6.9%) (6.71%, 7.05%)	924 / 5,978 (15.5%) (14.6%, 16.4%)
Hispanic		4,917 (5.7%) (5.50%, 5.81%)	643 / 4,917 (13.1%) (12.2%, 14.1%)
Multiple		3,267 (3.8%) (3.63%, 3.89%)	423 / 3,267 (12.9%) (11.8%, 14.2%)
Other		1,122 (1.3%) (1.22%, 1.37%)	178 / 1,122 (15.9%) (13.8%, 18.2%)
**Age**	86,954		
Under 25		2,344 (2.7%) (2.59%, 2.81%)	232 / 2,344 (9.9%) (8.73%, 11.2%)
25–34		8,803 (10.1%) (9.92%, 10.3%)	1,099 / 8,803 (12.5%) (11.8%, 13.2%)
35–44		11,088 (12.8%) (12.5%, 13.0%)	1,590 / 11,088 (14.3%) (13.7%, 15.0%)
45–54		12,327 (14.2%) (13.9%, 14.4%)	1,966 / 12,327 (15.9%) (15.3%, 16.6%)
55–64		18,339 (21.1%) (20.8%, 21.4%)	2,880 / 18,339 (15.7%) (15.2%, 16.2%)
65+		34,053 (39.2%) (38.8%, 39.5%)	4,521 / 34,053 (13.3%) (12.9%, 13.6%)
**HHI ($)**	86,954		
None		4,056 (4.7%) (4.53%, 4.81%)	962 / 4,056 (23.7%) (22.4%, 25.1%)
1k-19k		7,377 (8.5%) (8.30%, 8.67%)	1,722 / 7,377 (23.3%) (22.4%, 24.3%)
20k-49k		14,593 (16.8%) (16.5%, 17.0%)	2,565 / 14,593 (17.6%) (17.0%, 18.2%)
50k-99k		26,236 (30.2%) (29.9%, 30.5%)	3,649 / 26,236 (13.9%) (13.5%, 14.3%)
100k-149k		16,174 (18.6%) (18.3%, 18.9%)	1,745 / 16,174 (10.8%) (10.3%, 11.3%)
150k+		18,518 (21.3%) (21.0%, 21.6%)	1,645 / 18,518 (8.9%) (8.48%, 9.30%)
**Degree**	86,954		
No College Degree		28,860 (33.2%) (32.9%, 33.5%)	5,380 / 28,860 (18.6%) (18.2%, 19.1%)
College Degree		58,094 (66.8%) (66.5%, 67.1%)	6,908 / 58,094 (11.9%) (11.6%, 12.2%)
**Home Ownership**	86,954		
Rent		26,560 (30.5%) (30.2%, 30.9%)	4,801 / 26,560 (18.1%) (17.6%, 18.5%)
Own		60,394 (69.5%) (69.1%, 69.8%)	7,487 / 60,394 (12.4%) (12.1%, 12.7%)
		^*1*^ n (%)	^*3*^ n / N (%)
		^*2*^ CI = Confidence Interval	^*4*^ CI = Confidence Interval

### Response feature operationalization

Our study considers the outcome Major Depressive Disorder (MDD). 392,260 participants contributed electronic health records to All of Us, which populate the medical concepts database. We use the medical concepts “Major depressive disorder” (SNOMED code 370143000) as listed in the All of Us data browser. In this approach, we follow Barr and colleagues [34]. Their comparison utilizes phecodes available in All of US to label diagnoses. As explained in their measures section, diagnoses for disorders were based on phecodes derived from billing codes of the International Statistical Classification of Diseases, Ninth and Tenth Revisions, Clinical Modification (ICD-9/10-CM). Individuals with 2 or more phecodes from the selected CD-9/10-CM codes under the broader categories of drug-related disorders, mental disorders, substance abuse, sleep disorders, and mental state, were considered to have a diagnosis.

Of those contributing electronic health records with at least one condition recorded in the database, 26.2% recorded the MDD SNOMED code in their records. The MDD code refers to a hierarchy of conditions that includes single episodes of major depression, recurrent major depression, major depression in remission, minimal major depression, mild major depression, moderate major depression, severe major depression, major depression with psychotic features, and melancholic-type major depression. Our analytical framework organizes the data matrix into individual records corresponding to participants and variables representing each feature or outcome label. Within this framework, MDD status is binary-encoded.

### Demographic feature operationalization

All demographic features were sourced from The Basics survey, which all participants are required to complete. Six factors were selected for our analysis: gender/sexual identity, race/ethnicity, household income, age, education level, and home ownership. These categories are further decomposed into the following features:

1. Gender/sexuality: cisgender heterosexual male, cisgender heterosexual female, LGBTQIA2+
2. Race/ethnicity: White, Hispanic/Latinx, Black or African American, Asian, more than one population, or other
3. Age: continuous (units = Years)
4. Income: continuous (units = $10,000)
5. Education: college degree, no college degree
6. Home ownership: homeowner, not a homeowner

The six demographic labels are factorized into their respective categories. To prevent collinearity, each label is designated a reference category, which is omitted from the feature set for regression. After removing the reference features from the feature set, we are left with eleven demographic features.

For all the above fields, if an individual recorded a skipped question, preferred not to answer, or otherwise did not answer a question, they are not included in the analytical sample. More detailed information about how demographic features were operationalized in available in Box C of S1 Text. More detailed demographic distributional information is presented in Table B in S1 Text.

### SDoH feature operationalization

All of Us Social Determinants of Health Survey questions were aggregated from several validated instruments designed to measure specific social determinants of health. However, health care access and coverage are not captured in the All of Us Social Determinants of Health survey. Instead, these items are covered in The Basics and Health Care Access and Utilization surveys, which were administered before the Social Determinants of Health Survey. Informed by a review of extant SDoH literature [13,40–43] and the AoU design, we combined selected healthcare questions from these other surveys with the Social Determinants of Health survey to form the corpus of questions for our analysis of social determinants of health. These questions are partitioned into ten categories.

We partitioned the Social Determinants of Health Survey questions by their source instrument and treated each source instrument as a unique social determinant of health field. However, to mitigate issues due to collinearity, some source instruments were excluded because of thematic overlap with other instruments. The Discrimination in Medical Settings survey was removed due to its similarity to the Everyday Discrimination survey. Additionally, we empirically found answers to the RAND MOS Social Support Survey Instrument to be highly correlated with answers to the UCLA Loneliness Scale. Accordingly, we removed the RAND MOS Social Support Survey. The Cohen’s Perceived Stress Scale was also dropped because of its similarity to anxiety screening tools and the comorbidity of anxiety disorders and MDD. The English proficiency questions from the California Health Interview Survey were also dropped because there were few non-fluent English speakers in our analytical sample. Lastly, the Brief Multidimensional Measure of Religiousness/Spirituality survey was dropped because religion is rarely included as a social determinant of health. In summary, we use ten social determinants of health features.

1. Food Security
2. Discrimination
3. Neighborhood Disorder
4. Neighborhood Social Cohesion
5. Neighborhood Infrastructure/Facilities
6. Number of Moves in the Past 12 Months
7. Loneliness
8. Housing Issues
9. Lack of Health Care Access
10. Health Insurance

There are four different encoding types for the social determinants of health fields: mean subscales, numeric responses, sum of checked responses, and indicator; see Table 2. These are further described in the Box D in S1 Text.

**Table 2:** Summary of SDoH factors with source, encoding, and number of items.

SDoH Field	AoU Survey	Encoding Type	# Items
Food Insecurity	Social Determinants of Health	Mean Subscale	2
Discrimination	Social Determinants of Health	Mean Subscale	9
Neighborhood Disorder	Social Determinants of Health	Mean Subscale	13
Neighborhood Social Cohesion	Social Determinants of Health	Mean Subscale	4
Neighborhood Infrastructure	Social Determinants of Health	Mean Subscale	7
Loneliness	Social Determinants of Health	Mean Subscale	8
Transience	Social Determinants of Health	Numeric Response	1
Housing Issues	Social Determinants of Health	Sum of Checked Responses	1
Lack of Health Care Access	Health Care Access & Utilization	Sum of Checked Responses	1
Health Insurance	The Basics	Indicator	1

## Procedure and analysis strategy

We use a staged multiple logistic regression design. In the first stage, we consider how demographic factors independently predict risk for diagnosis of MDD. In this stage, the feature set consists of only the eleven demographic features. In the second stage, we consider how social determinants of health add information toward prediction of MDD diagnosis. In this stage, the feature set consists of the 11 demographic features and the 10 social determinants of health features. In the third stage, we consider how select demographic factors moderate the role of social determinants of health in predicting MDD diagnosis. In this stage, the base feature set consists of the union of demographic and social determinants of health features, of size 21. We choose to focus on race/ethnicity and gender/sexual identity as demographic moderators. We also exclude the age, income, education, home ownership, health care access, and health insurance from consideration as moderation effects, instead interpreting these as controls, as access to health care and availability of health insurance are bottlenecks to receiving a diagnosis of MDD. We then consider pairwise interaction terms between selected demographic moderators and social determinants of health. In RQ3, we choose to focus on the correlational moderating effects of gender/sexual identity and race/ethnicity on food insecurity, discrimination, neighborhood social cohesion, and loneliness. The set of interaction terms examined in RQ3 is of size 28. We further use expected marginal means interaction analysis to isolate specific interaction effects and corroborate findings from the regression models. Table 3 summarizes our modeling approach and the features used in each stage of our analysis.

**Table 3:** Feature sets.

Dependent Variables
Indication (0/1) for each of: • Major Depressive Disorder (MDD)
Stage 1: Demographics–Research Question 1
• Age (Continuous, Years) • HHI (Continuous, $10k) • Education (Reference = College Degree or More, 2 levels) • Home Ownership (Reference = Own, 2 levels) • Gender/Sexual Identity (Reference = Cisgender Heterosexual Male, 3 levels) • Minority Race/Ethnicity (Reference = White Non-Hispanic, 6 levels)
Stage 2: Social Determinants of Health—Research Question 2
• Food Security (Calculated factor scaled between 0–1) • Discrimination (Calculated factor scaled between 0–1) • Neighborhood Disorder (Calculated factor scaled between 0–1) • Neighborhood Social Cohesion (Calculated factor scaled between 0–1) • Neighborhood Infrastructure/Facilities (Calculated factor scaled between 0–1) • Number of Moves in Past 12 Months (Transience) (Calculated factor scaled between 0–1) • Loneliness (Calculated factor scaled between 0–1) • Housing Issues (Calculated factor scaled between 0–1) • Health Care Access (Calculated factor scaled between 0–1) • Health Insurance (Calculated factor scaled between 0–1)
Stage 3: Select Social Position Moderators—Research Question 3
• Food Insecurity X: ○ Gender/Sexual Identity (Reference = Cisgender Heterosexual Male) ○ Minority Race/Ethnicity (reference = White Non-Hispanic) • Discrimination X: ○ Gender/Sexual Identity (Reference = Cisgender Heterosexual Male) ○ Minority Race/Ethnicity (reference = White Non-Hispanic) • Neighborhood Social Cohesion X: ○ Gender/Sexual Identity (Reference = Cisgender Heterosexual Male) ○ Minority Race/Ethnicity (reference = White Non-Hispanic) • Loneliness X: ○ Gender/Sexual Identity (Reference = Cisgender Heterosexual Male) ○ Minority Race/Ethnicity (reference = White Non-Hispanic)

## Results

Table 4 reports an overview of the results of hierarchal logistic regression model. The table includes fit statistics for each stage of the model as information is added to the hierarchy. The Akaike information criteria (AIC) for each model, the change in the *Χ*^2^ statistic as information is added, and the significance of the change in model fit are presented. We find that each stage of the modeling significantly improves fit. These stages include the addition of demographic controls; gender identity and sexual orientation and race and ethnicity; the SDoH factors; and the select moderation effects. Broadly, Table 4 affirms that, controlling for age and income, gender identity/sexual orientation and race/ethnicity are significant predictors of depression (RQ1). SDoH (RQ2) and the select moderations examined in RQ3 are also significant predictors of depression.

**Table 4:** Hierarchical modeling summary.

Model Stage	df	AIC	Δ *Χ*^2^	Δ *Χ*^2^ df	Sig.
**Intercept**	1	70842.63			
**+ Demographic Controls**	5	69221.79	1628.83	4	<0.001
**+ GISO + Race/Ethnicity**	12	68536.74	699.05	7	<0.001
**+ SDoH**	22	67181.75	1374.99	10	<0.001
**+ Moderation**	50	67106.24	131.51	28	<0.001

Degrees of freedom (df), Akaike information criteria (AIC), Chi-squared (*Χ*^2^).

Table 5 provides more detailed information about how these predictors are related to risk of depression. We include the adjusted odds ratio (AOR) as a measure of the effect on the risk of depression, an associated 95% confidence interval (CI) to emphasize in our estimate, and the achieved significance (Sig) of the estimate. Specifically, Table 5 reports the results for Stage 1, demographics; Stage 2 (+ SDoH), and Stage 3 (+ select moderations for RQ3).

**Table 5:** Detailed model results.

	Stage 1 (Demographics)			Stage 2 (+ SDoH)			Stage 3 (+ Select Moderation)		
Characteristic	AOR	95% CI^*1*^	Sig.	AOR	95% CI^*1*^	Sig.	AOR	95% CI^*1*^	Sig.
Demographics									
**HHI ($10k)**	1.00	1.0, 1.00	<0.001	1.00	1.00, 1.00	<0.001	1.00	1.00, 1.00	<0.001
**Age (Years)**	1.01	1.00, 1.01	<0.001	1.01	1.01, 1.01	<0.001	1.01	1.01, 1.01	<0.001
**No College Degree**	1.33	1.28, 1.39	<0.001	1.30	1.25, 1.36	<0.001	1.31	1.25, 1.37	<0.001
**Rent Home**	1.30	1.24, 1.37	<0.001	1.18	1.12, 1.24	<0.001	1.17	1.12, 1.23	<0.001
**Gender and Sexual Identity**									
** CH male**	—	—		—	—		—	—	
** CH female**	1.60	1.53, 1.68	<0.001	1.54	1.47, 1.61	<0.001	2.14	1.63, 2.81	<0.001
** GSM**	1.71	1.60, 1.84	<0.001	1.50	1.39, 1.61	<0.001	2.80	1.91, 4.11	<0.001
**Race/Ethnicity**									
** White**	—	—		—	—		—	—	
** Asian**	0.41	0.35, 0.49	<0.001	0.42	0.35, 0.50	<0.001	0.10	0.03, 0.30	<0.001
** Black**	0.75	0.69, 0.81	<0.001	0.77	0.71, 0.84	<0.001	0.98	0.67, 1.41	0.893
** Hispanic**	0.66	0.60, 0.72	<0.001	0.74	0.68, 0.81	<0.001	0.79	0.51, 1.21	0.271
** Multiple**	0.79	0.71, 0.88	<0.001	0.76	0.69, 0.85	<0.001	0.77	0.43, 1.37	0.370
** Other**	1.00	0.85, 1.18	>0.900	0.92	0.78, 1.09	0.400	0.82	0.33, 2.01	0.662
**SDoH**									
** Food Insecurity**				1.30	1.17, 1.44	<0.001	1.41	1.11, 1.80	0.005
** Discrimination**				1.40	1.22, 1.61	<0.001	1.40	1.05, 1.86	0.021
** Neighborhood Disorder**				0.95	0.83, 1.10	0.500	0.95	0.82, 1.09	0.441
** Social Cohesion**				0.92	0.81, 1.05	0.200	0.86	0.66, 1.10	0.226
** Neighborhood Infrastructure**				1.11	1.01, 1.22	0.030	1.10	1.00, 1.21	0.055
** Housing Transience**				0.95	0.92, 0.98	<0.001	0.95	0.92, 0.98	<0.001
** Loneliness**				6.89	6.04, 7.87	<0.001	14.0	10.7, 18.4	<0.001
** Healthcare Access**				1.30	1.16, 1.46	<0.001	1.34	1.19, 1.50	<0.001
** Have Health Insurance**				1.58	1.38, 1.82	<0.001	1.61	1.40, 1.85	<0.001
** Home Structure Problems**				0.97	0.82, 1.14	0.700	0.99	0.84, 1.17	0.903
**Select Moderation**									
Gender and Sexual Identity * Food Insecurity									
CH female * Food Insecurity							0.98	0.75, 1.26	0.847
GSM * Food Insecurity							0.60	0.43, 0.83	0.002
Race/Ethnicity * Food Insecurity									
Asian * Food Insecurity							0.62	0.20, 1.92	0.404
Black * Food Insecurity							1.05	0.80, 1.38	0.728
Hispanic * Food Insecurity							1.32	0.94, 1.83	0.105
Multiple * Food Insecurity							0.99	0.64, 1.52	0.950
Other * Food Insecurity							1.74	0.90, 3.37	0.097
Gender and Sexual Identity * Discrimination									
CH female * Discrimination							0.98	0.71, 1.35	0.908
GSM * Discrimination							0.99	0.65, 1.51	0.957
Race/Ethnicity * Discrimination									
Asian * Discrimination							2.43	0.73, 8.12	0.148
Black * Discrimination							1.32	0.90, 1.95	0.157
Hispanic * Discrimination							0.77	0.45, 1.29	0.314
Multiple * Discrimination							1.15	0.62, 2.11	0.658
Other * Discrimination							0.60	0.22, 1.62	0.310
Gender and Sexual Identity * Social Cohesion									
CH female * Social Cohesion							1.11	0.84, 1.47	0.467
GSM * Social Cohesion							1.37	0.92, 2.04	0.120
Race/Ethnicity * Social Cohesion									
Asian * Social Cohesion							4.26	1.40, 13.0	0.011
Black * Social Cohesion							0.75	0.50, 1.10	0.144
Hispanic * Social Cohesion							0.72	0.45, 1.15	0.167
Multiple * Social Cohesion							0.95	0.52, 1.74	0.874
Other * Social Cohesion							1.13	0.45, 2.87	0.797
Gender and Sexual Identity * Loneliness									
CH female * Loneliness							0.43	0.32, 0.59	<0.001
GSM * Loneliness							0.23	0.14, 0.35	<0.001
Race/Ethnicity * Loneliness									
Asian * Loneliness							1.64	0.50, 5.37	0.411
Black * Loneliness							0.75	0.47, 1.18	0.213
Hispanic * Loneliness							1.33	0.76, 2.35	0.319
Multiple * Loneliness							1.00	0.50, 1.98	0.994
Other * Loneliness							1.19	0.41, 3.49	0.753
^*1*^ CI = Confidence Interval									

Table 5 shows the main effects to address RQ1 and RQ2 as well as the interactions terms included in our analysis to assess the moderation effects associated with RQ3. Abbreviations: CH = Cisgender Heterosexual, GSM = Gender/Sexual Minority (i.e., Lesbian, Gay, Bisexual, Transgender, Queer, Intersex, Asexual, Two Spirit, and additional minoritized gender and sexual identities (LGBTQIA2+)). AOR = Adjusted Odds Ratio, CI = Confidence Interval, and Sig. = Significance.

Although, as shown in Table 4, all phases of the regression significantly improve the model, few parameter estimates change substantively as new information is added. For example, between Stages 1 and 2, there are no changes to the significance of the demographic factors when the SDoH factors are added as main effects. When moderation is included in Stage 3, the significance of race/ethnicity decreased, consistent with the reallocation of predictive power from the main effect to the moderating interaction.

We followed Johnson (2013) in our selection 0.005 significance level due to the size of our analytical sample and the number of effects being estimated in our model [44]. For the sake of parsimony, we focus our discussion on parameter estimates that are significant at 0.005 or below in the Stage 3 model as well as estimates predicted to be significant in the literature but for which our results failed to find similar results at 0.005. Specifically, see Discrimination in Table 5 with an AOR of 1.40, significant at 0.021. As noted in our literature review, discrimination is implicated in other mental health studies in complex multifactorial ways. Similarly, we make note of the effect associated with Asian participants and the SDoH Social Cohesion (AOR = 4.26, p = 0.011) because of the large size of the effect and its implication. Namely, increased social cohesion appears to be associated with an increased likelihood of depression, a result that deserves further research, as we discuss below.

### Demographic main effects

Compared to Cisgender Heterosexual Males, Cisgender Heterosexual Females and LGBTQIA2+ individuals are more than twice as likely to have an MDD diagnosis (AOR = 2.14 and 2.80, respectively, p < 0.001). Other social demographic factors also predict MDD. Less education (no college degree) and renting one’s home, respectively, also predict an MDD diagnosis compared to more education and owning a home (AOR = 1.31 and 1.17, respectively, p < 0.001). In our analysis, only the Asian/Asian American race/ethnicity category differs significantly from White individuals, with Asian/Asian American community members having an AOR = 0.10, p < 0.001. The AOR for MDD for all other race/ethnicity categories are statistically indistinguishable from that of White individuals at any reasonable level of significance. In summary, relative to RQ1, our analysis shows that sexual and gender identity are strongly predictive of MDD, but that race and ethnicity are much less predictive of MDD.

### SDoH main effects

Higher Food Insecurity (AOR = 1.41, p = 0.005) and greater Loneliness (AOR = 14.05, p <0.001) both indicate a higher likelihood of MDD. With a significance of 0.021, discrimination did not meet our threshold for significance, but had an AOR of 1.40, an indication of higher likelihood of MDD as found in the previous studies covered in our literature review. Transience (AOR = 0.95, p < 0.001), Lack of Health Care Access (AOR = 1.34, p < 0.001), and having Health Insurance (AOR = 1.61, p < 0.001) also significantly predict MDD diagnosis. Transience, with an AOR less than one, reduced the likelihood of having an MDD diagnosis; the other significant factors just mentioned increase the likelihood of an MDD diagnosis. In summary, relative to RQ2, our analysis indicates that Food Insecurity and Loneliness, as expected, are substantially connected to increased likelihood of MDD. Social Cohesion was not a significant predictor of MDD.

### Moderation effects

Our analysis for RQ3 focuses on how race/ethnicity and gender and sexual identity interact with Food Insecurity, Discrimination, Neighbor Social Cohesion, and Loneliness to assess to what degree these four SDoH factor have differential impact on MDD for different identities. The interaction between Food Insecurity and Gender and Sexual Identity revealed a reduction in the likelihood of an MDD diagnosis for GSM individuals (AOR = 0.60, p = 0.002). The interaction between Discrimination and Race/Ethnicity was not significant at 0.005. The interaction between the SDoH Neighborhood Social Cohesion and the Asian race produced a significant result (AOR = 4.26, p = 0.011). We make note of this result even though it exceeds our 0.005 cutoff because the main effect for Asian individuals compared to White individuals was highly significant (AOR = 0.10, p < 0.001). Together these effects point to some of the complexity in the linkage between demographic identities and SDoH. Here, Asian individuals have a lower likelihood than White individuals, but there is some indication of a moderation effect with increased social cohesion that increases the likelihood of depression as social cohesion increases. Last, the interaction between Loneliness and Gender and Sexual identity revealed significant reductions in the likelihood of MDD in the interaction terms for both Cisgender Heterosexual Females and GSM individuals, compared to Cisgender Heterosexual Males, with respective AOR values of 0.43 and 0.23, p < 0.001 for both. In summary, relative to RQ3, our analysis produced mixed support for differential SDoH effects on MDD for identities defined by race/ethnicity and gender/sexual orientation.

**Fig 1:**
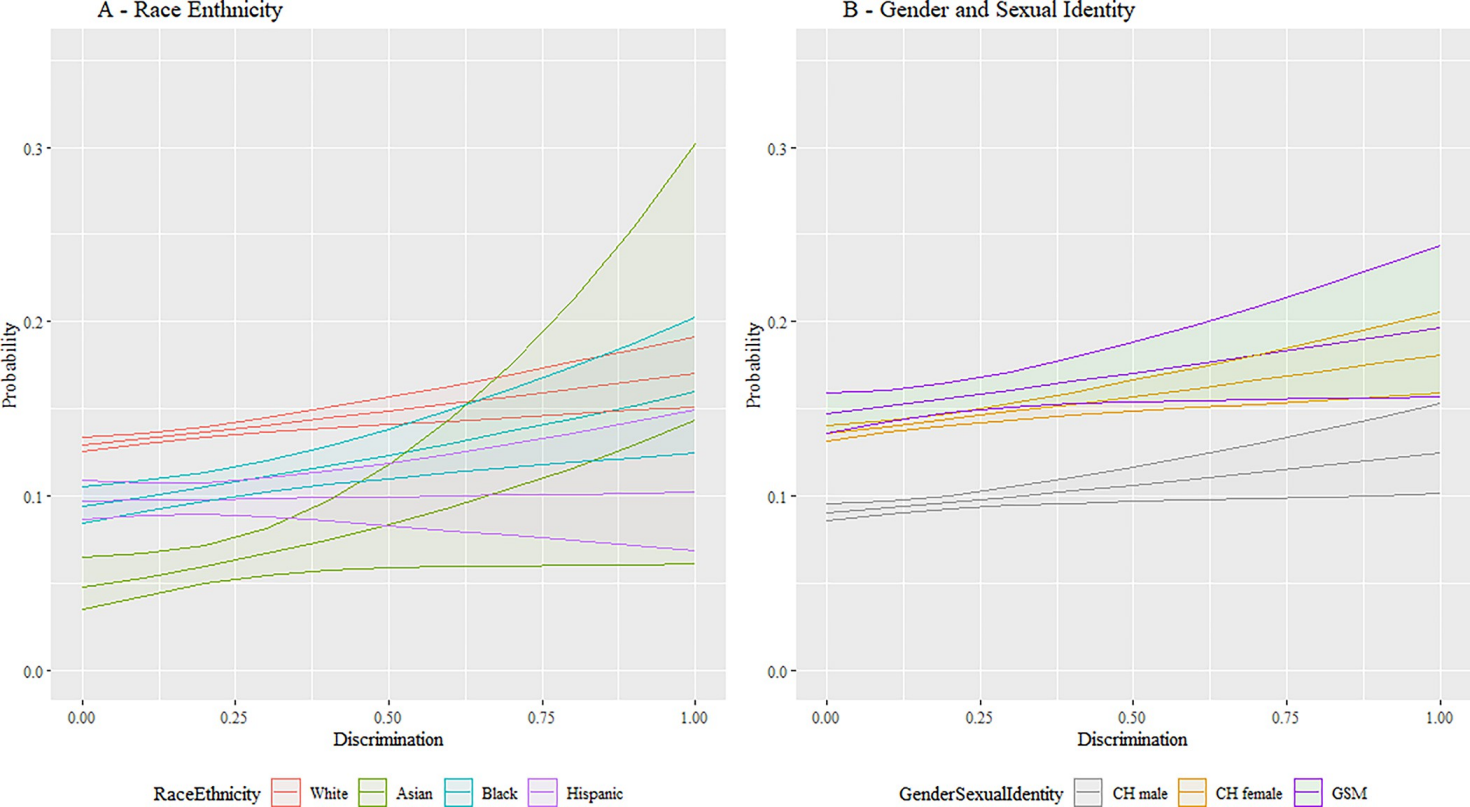
Interaction plot for discrimination. Fig 1 shows the interaction between the SDoH factor for discrimination versus the demographic factors for race and ethnicity and gender and sexual identity. The vertical axis is the predicted likelihood of an AoU participant in our analytical sample experiencing MDD. The horizontal axis for discrimination has the range [0,1], where a higher value corresponds with a participant’s neighborhood having more discrimination. Abbreviations: CH = Cisgender Heterosexual, GSM = Gender/Sexual Minority (i.e., Lesbian, Gay, Bisexual, Transgender, Queer, Intersex, Asexual, Two Spirit, and additional minoritized gender and sexual identities (LGBTQIA2+)). 95% confidence bands are included.

**Fig 2:**
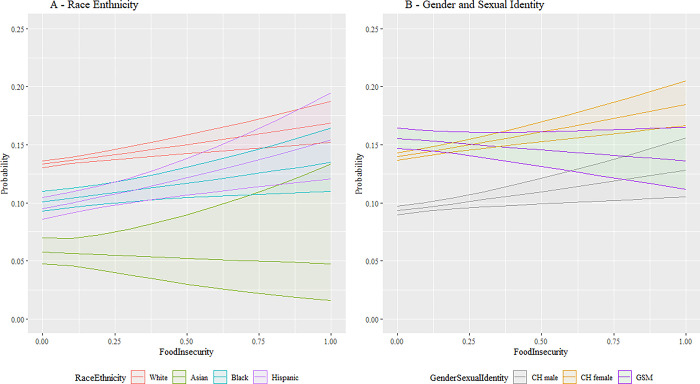
Interaction plot for food insecurity. Fig 2 shows the interaction between the SDoH factor for food insecurity versus the demographic factors for race and ethnicity and gender and sexual identity. The vertical axis is the predicted likelihood of an AoU participant in our analytical sample experiencing MDD. The horizontal axis for food insecurity has the range [0,1], where a higher value corresponds with a participant having more food insecurity. Abbreviations: CH = Cisgender Heterosexual, GSM = Gender/Sexual Minority (i.e., Lesbian, Gay, Bisexual, Transgender, Queer, Intersex, Asexual, Two Spirit, and additional minoritized gender and sexual identities (LGBTQIA2+)). 95% confidence bands are included.

**Fig 3:**
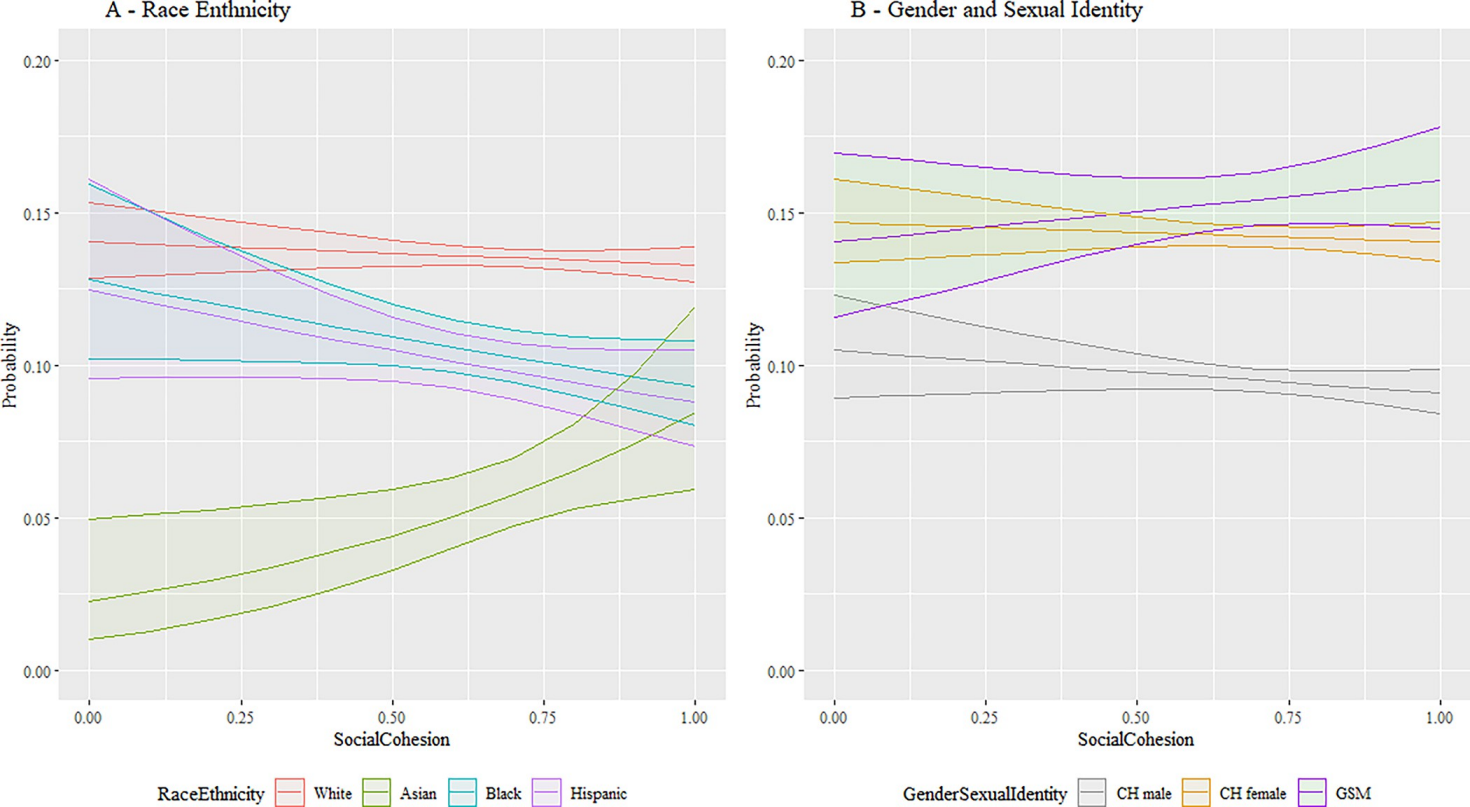
Interaction plot for social cohesion. Fig 3 shows the interaction between the SDoH factor for social cohesion versus the demographic factors for race and ethnicity and gender and sexual identity. The vertical axis is the predicted likelihood of an AoU participant in our analytical sample experiencing MDD. The horizontal axis for social cohesion has the range [0,1], where a higher value corresponds with a participant having more social cohesion. Abbreviations: CH = Cisgender Heterosexual, GSM = Gender/Sexual Minority (i.e., Lesbian, Gay, Bisexual, Transgender, Queer, Intersex, Asexual, Two Spirit, and additional minoritized gender and sexual identities (LGBTQIA2+)). 95% confidence bands are included.

Next, we employ expected marginal means and interaction plots to support a deeper conceptual review of these analytical findings with a more accessible presentation. This approach allows the effects of the various parameter estimates associated with our research questions to be highlighted by combining the main and interaction effects from predictors added, respectively, in stages 1, 2, and 3 while averaging across all other variables in the model. Fig 1 combines these effects visually for discrimination to illustrate the differences between racial and ethnic groups as well as between gender and sexual identities. Differences in base rate, where perceived discrimination is low, are apparent. As perceived discrimination increases, vastly different slopes are apparent until low sample power increases the variability to the extent that confidence intervals on the prediction overlap. Similar patterns are seen in Fig 2 for food insecurity. Fig 3 shows similar relationships; here White, Black, and Hispanic individuals as well as CH males and CH females, increased social cohesion reduces the probability of depression. This protective effect is not shared by, respectively, Asian and GSM individuals.

**Fig 4:**
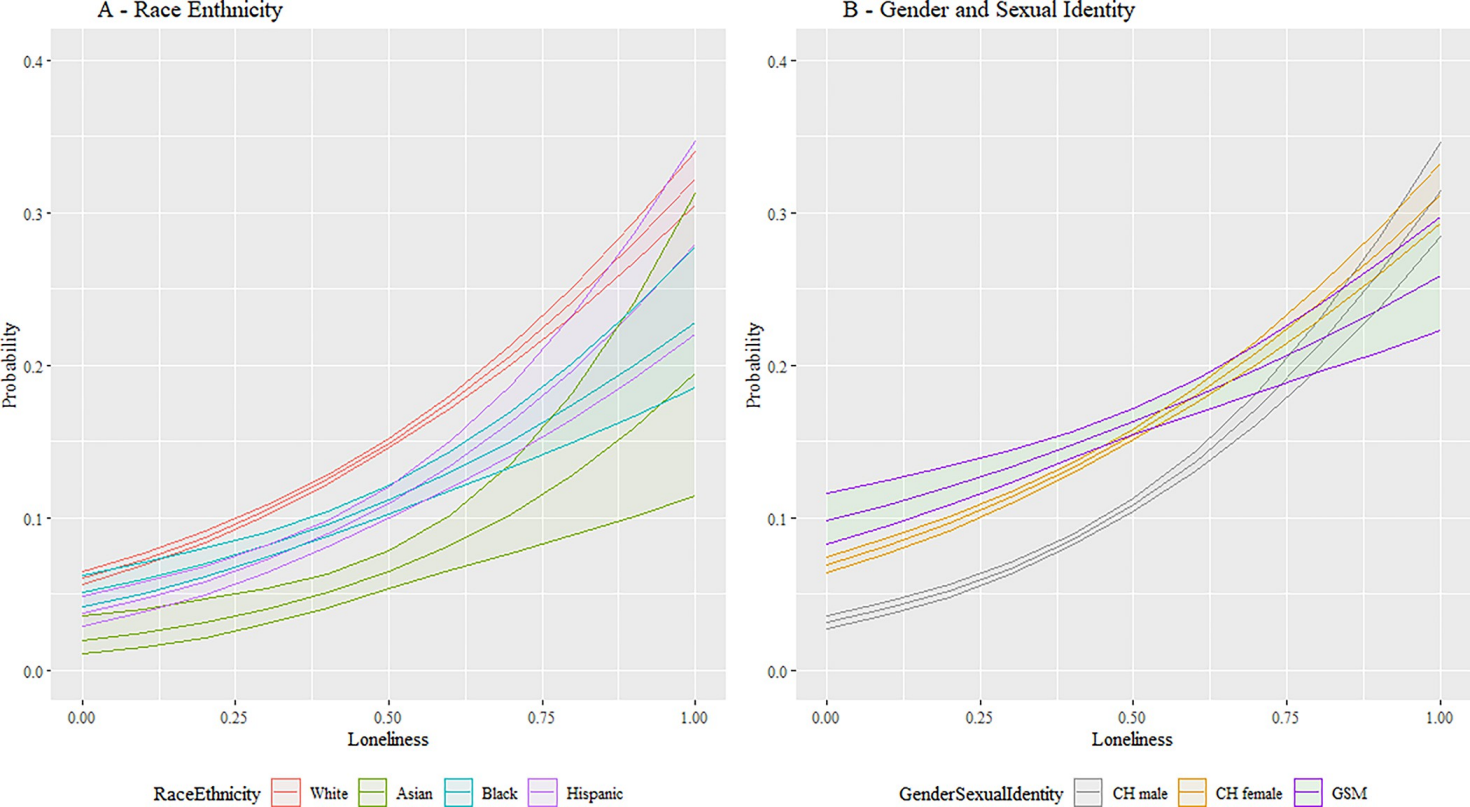
Interaction plot for loneliness. Fig 4 show the interaction between the SDoH factor for loneliness versus the demographic factors for race and ethnicity and gender and sexual identity. The vertical axis is the predicted likelihood of an AoU participant in our analytical sample experiencing MDD. The horizontal axis loneliness has the range [0,1], where a higher value corresponds with a participant having more loneliness. Abbreviations: CH = Cisgender Heterosexual, GSM = Gender/Sexual Minority (i.e., Lesbian, Gay, Bisexual, Transgender, Queer, Intersex, Asexual, Two Spirit, and additional minoritized gender and sexual identities (LGBTQIA2+)). 95% confidence bands are included.

Fig 4 illustrates that, as expected, loneliness is a substantial driver of depression, with increases to loneliness significantly increasing the likelihood of an MDD diagnosis as a main effect. This corresponds to the AOR = 14.0 (p < 0.001) in Table 5. Moreover, our results indicate that loneliness affects cisgender heterosexual female community members and gender and sexually minoritized community members more weakly than cisgender heterosexual males (AOR = 0.43 and 0.23, p < 0.001, respectively in Table 5). However, Fig 4 makes clear that including main effects and moderation, all race and ethnic groups as well as all gender and sexual identities are profoundly impacted loneliness.

## Discussion

Extant research has examined the roles of various demographic characteristics and social determinants of health on mental health outcomes. We contribute to this literature by focusing on major depressive disorder (MDD) and investigating how race, ethnicity, gender, and sexual identity moderate the influence of several SDoH domains on this common mental health condition. Our findings highlight the intricate and nuanced ways in which the context in which people live their lives significantly impacts health outcomes. Additionally, our discussion suggests directions for future research.

Consistent with prior research, our Stage 3 results reveal significant main effects that contribute to MDD risk. Specifically, women and LGBTQIA2+ individuals exhibit a significantly higher likelihood of MDD diagnosis compared to cisgender heterosexual males [12,16,17]. This underscores the substantial influence of gender and sexual identity on MDD. Moreover, less education (lacking a college degree) and renting one’s home also predict a higher likelihood of MDD diagnosis, emphasizing the importance of socio-economic factors [23]. Interestingly, our study also reveals a lower likelihood of MDD diagnosis among Asian/Asian American individuals compared to White individuals. While this finding contrasts with some literature that emphasizes racial and ethnic disparities in mental health outcomes, it underscores the complexity of the relationship between race, ethnicity, and MDD risk [12].

Although the main effects of race and ethnicity on MDD risk appears less pronounced in our Stage 3 results, we note that they are very strong in Stage 2 results. When controlling for social determinants of health and other demographic factors, we see that White community members have a significantly higher likelihood of MDD diagnosis than all other groups. Further, Asian community members are 58% less likely than White individuals to be diagnosed with MDD, Black community members are 23% less likely to be diagnosed with MDD, Hispanic community members are 26% less likely to be diagnosed with MDD, and individuals who identify with multiple races/ethnicities are 24% less likely to be diagnosed with MDD. However, the relationship between race and MDD diagnosis changes when moderation effects are included in Stage 3. All the aforementioned reductions in risk of MDD among people of color compared to White individuals become insignificant except for Asian individuals, indicating the differential likelihood of MDD diagnosis between people of color and White individuals is largely attributed to the unique moderation effects of social determinants of health on these two groups. It is important to reiterate that our analysis of MDD risk is based on a confirmed MDD diagnosis rather than self-report or perception of MDD. The low likelihood of MDD diagnosis among Asian Americans can partly be attributed to disparities in mental healthcare utilization between White and Asian individuals, respectively, even controlling for perceived need [45]. Because African American and Latino individuals, for example, are more likely to lack access to mental healthcare compared to White individuals, the likelihood of receiving an MDD diagnosis may be lower. As a result, the prevalence of the disorder among some populations may be undercounted due to factors like these.

Regarding the influence of SDoH factors on MDD risk, our results align with previous research indicating higher levels of food insecurity and loneliness are strongly associated with an increased likelihood of MDD [23]. The strong associations between these factors and MDD underscore the importance of addressing social and environmental determinants of mental health. However, our finding that social cohesion does not emerge as a significant predictor contradicts some literature emphasizing the protective role of neighborhood cohesion against psychological distress [24]. This inconsistency highlights the need for further investigation into the complex relationships between social support, community factors, and mental health outcomes.

Additionally, while discrimination did not meet our significance threshold as a predictor of MDD risk, its noteworthy effect in the literature suggests the need for continued attention to the impact of stigma and discrimination on mental health disparities [46]. Similarly, our finding that transience reduces the likelihood of MDD diagnosis contradicts conventional wisdom and warrants further exploration [25].

While few moderation effects in the Stage 3 model reached our threshold for significance, those that do shed light on less-established moderation effects or offer new perspectives on well-studied interactions. Although we found loneliness to be a driver of depression across population segments, our analysis indicates loneliness affects cisgender heterosexual women and gender and sexually minoritized community more weakly than cisgender heterosexual men. This interaction effect has been documented in prior research. Cacioppo et al. (2002) found, in a nationally representative sample of Americans aged 54 or older, that loneliness increased the number of depressive symptoms in men at a greater rate than women [47]. However, a 2018 study of 168 African-American college students found, inversely, that loneliness decreased depressive symptoms in women more than men [48]. The majority of respondents in our analytical sample are aged 60 or older, so our sample bears similarity to the one used in Cacioppo et al., and has particularly poor representation of college-aged African American population considered in Chang’s work. Our analysis reinforces that loneliness is a greater risk factor toward depression in men than women among a majority retirement-aged population, and also shows this the case in men with respect to GSM individuals, but further research is needed to determine to what degree this relationship generalizes across populations, and for which groups women and GSM individuals are more adversely affected by loneliness than cisgender heterosexual men.

A less studied relationship is the interaction between Asian race/ethnicity and social cohesion toward MDD diagnosis. Our analysis indicates that while Asians are far less likely to experience depression (AOR = 0.10, p < 0.001), greater social cohesion increases the likelihood of a depression diagnosis among Asians (AOR = 4.26). This is the only racial/ethnic group in our analysis for which this is true. One hypothesis is that both depression and mental health resource utilization can be modeled as a social network phenomena [49,50] and these network effects are differentially expressed across different population segments. However, it is difficult to attribute the moderating effect of race/ethnicity on social cohesion to any single cause, and analyses with greater statistical power are necessary to determine the robustness of this effect before further speculation is warranted. This underscores the complexities in the interaction of socioeconomic factors and SDoH, particularly in how they are manifested and measured within the diverse AoU sample.Our study delves into the complex interplay between demographic factors and SDoH, suggesting that this complexity extends beyond traditional demographics to a broader consideration of social position (SP).The relationships between objective sociodemographic factors and subjective assessments of social position (SP) have been studied without universal agreement on definitions; see Alder and colleagues (2000) for a discussion [51]. Lindemann (2007) examined an individual’s perceived place in society, or social position, and the influence of observable variables like age, gender, ethnicity, education, employment, and income. As Lindemann states, “subjective social position depends not only on the objective characteristics but also on how people experience society, the way they perceive their position in comparison with others, and what they imagine their position would be in future.” [52] In this regard, our findings indicate a weaker than expected influence of straightforward SDoH interactions, which may reflect limitations within the AoU sample composition that do not fully capture the nuanced ways in which socioeconomic factors interact with health outcomes. This highlights the need for a broader array of measures to understand the full impact of SP on health, suggesting future research should expand beyond the regression analysis of the current study to include structural equation models that encompass mediation and moderation effects of SP on SDoH and their interacting roles on mental health outcomes. In the current study, we followed the lead of Avlund et al. (2003), who included education, occupation, social class, income, and housing tenure as measures of social position in a study of depression, finding strong associations between social position and measures of health [53]. Future research, and data collection by AoU, should include more comprehensive views of social position. The very barriers and gaps addressed by SDoH measurement appear to be intermediate outcomes of antecedent SP factors. Relatedly, future research should expand beyond the regression analysis of the current study to include structural equation models that encompass mediation and moderation effects of SP on SDoH and their interacting roles on mental health outcomes.

The All of Us dataset represents a valuable resource for the research community, mirroring social position representation that approximately reflects that of the US population overall. However, as our study reveals, different identity segments experience varying health outcomes based on their living context. To better understand the moderation effects of SDoH on social position in relation to health outcomes for marginalized communities, future research requires larger and more diverse samples. This is essential for improving our understanding of disease prevalence and associated factors, enabling the delivery of more relevant and culturally competent mental health care. While the All of Us dataset includes an expansive array of measurements that we have operationalized into a set of SDoH factors based on careful literature review and screening, alternative approaches for subtyping SDoH subdomains may exist. Future studies may benefit from exploring different operationalization strategies.

The All of Us Research Program includes an expansive array of measurements providing support for extremely detailed analysis of health outcomes and the drivers of those outcomes. However, participants who enroll in All of Us typically complete The Basics, Lifestyle, and Overall Health Surveys upon enrollment. The Social Determinants of Health is administered as an optional follow-up survey to All of Us participants. Further, the Social Determinants of Health survey has only been available since November 2021, making it the newest follow-up survey offered to participants. Therefore, the Social Determinants of Health survey was not offered to most participants upon enrollment. Only 117,800 of the 413,460 individuals who have participated in the All of Us program have taken the Social Determinants of Health survey. Individuals who did not participate in the Social Determinants of Health survey were omitted from our analytical sample. Some of those who did participate in the Social Determinants of Health Survey, however, recorded null responses that disqualified them from inclusion in our analytical sample. Our final analytical sample includes 86,960 participants.

The All of Us dataset includes an expansive array of measurements that we have operationalized into a set of SDoH factors, described in detail above. We acknowledge that while we based our operationalization strategy based on a careful review of extant literature and SDoH screeners [13,40–43] and the sources of the instruments from which AoU drew the questions, other choices might have been made. See Bhavnani and colleagues (2023) for another approach to subtyping SDoH subdomains from the measurements provided by AoU [54].

A lifespan approach is crucial to address the social determinants of mental health where improving mental health should start before birth and progress throughout one’s life. It highlights the importance of interventions during childhood, adolescence, family-building, working ages, and older age to reduce mental health disparities [55]. Similarly, Ploubidis et. al (2021) suggests that improving early-life mental health could have implications for population health and may help mitigate morbidity and mortality in later life. Effective interventions targeting early-life mental health have the potential to improve multiple physical health outcomes and reduce the risk of premature mortality [56].

## Limitations

The current study demonstrated the value of the AoU data in the study of how SDoH differentially drive health outcomes. It also provides a reminder that even larger datasets designed to represent the general population face substantial challenges for research focused on marginalized community segments and is a timely reminder that sampling plans are needed ensure sufficient statistical power to examine those most marginalized and underserved. Although adjacent to our focus on depression, it is worth noting that Barr et al. caution about the historical racial and diagnostic biases evident in the AoU data that skew, for example, schizophrenia diagnoses for people of color [34].

We also recognize that while we operationalized ten SDoH domains from the AoU data, the current study focused on only four of those domains in our examination of correlational moderating effects with demographics, primarily due to manuscript length constraints. Future research might probe other moderation and mediation relationships.

Despite these limitations, the All of Us Research Program provides a research asset that is without peer. Future planning for the All of Us data might consider oversampling of participants within select identity segments in addition to expanding the size of the general population sample. Oversampling within the intersections of race/ethnicity and gender/sexual identity is an example. More specifically, increasing the sample of women and nonbinary community members of color would allow studies to elucidate ways to improve the delivery of care to community members who endure disproportionate impact from several SDoH factors on, in the current study, major depressive disorder.

Lastly, while our study draws inspiration from intersectional frameworks, we acknowledge that it has limitations in the context of intersectionality theory. Specifically, our use of survey data and quantitative methods may not capture the more nuanced, anecdotal data points about an individual’s lived experience that a mixed methods study might. Further, while our framework illustrates the intersectional effects between *pairs* of identity groups, a more thorough intersectional analysis might investigate how *many* social dimensions intersect to contribute to health outcomes. Additionally, our use of linear models limits our intersection analysis to additive (main) effects and multiplicative (interaction) effects. The ability to identify non-linear relationships may make possible the analysis of a larger number of intersecting social dimensions. For example, Bauer et al. [57], in their systematic review of intersectionality in quantitative research, have suggested that non-linear models such as decision trees show promise for capturing the non-linear effects of a large number of interactions.

## Conclusions

The current study employs the All of Us Research Program dataset to unpack the potentially intersecting effects of various SDoH factors to examine the different roles they play on depression based on race, ethnicity, gender, and sexual identity. We employ staged logistic regression to examine main and moderating effects. Our analysis confirms the nuance and complexity of these relationships. We also use these analyses to outline future research to delve deeper into some of these findings. The current study demonstrated the value of the AoU data in the study of how various SDoH factors are differentially connected to health outcomes. It also provides a reminder that even larger datasets designed to represent the general population face substantial challenges for research focused on marginalized community segments and is a timely reminder that sampling plans are needed ensure sufficient statistical power to examine those most marginalized and underserved.

## Supporting information

S1 Text Box A in S1 Text—Connections between SDoH and Depression. Box B in S1 Text—All of Us Program Research Program Measurements. Table A in S1 Text—All of Us Research Program Survey Design. Box C in S1 Text—AoU Research Program Data Review, Demographic Feature Operationalization. Table B in S1 Text—All of US Research Program Detailed Demographics. Box D in S1 Text—Data Types used for SDoH Feature Operationalization.
(DOCX)
