# Supplementary material for: The role of social determinants of health in mental health: An examination of the moderating effects of race, ethnicity, and gender on depression through the all of us research program dataset
Source: PLOS Ment Health. 2024 Aug 5;1(3):e0000015. doi: 10.1371/journal.pmen.0000015 (PMC12798497; doi:10.1371/journal.pmen.0000015)
Supplement: S1 Text — Box B in S1 Text—All of Us Program Research Program Measurements. Table A in S1 Text—All of Us Research Program Survey Design. Box C in S1 Text—AoU Research Program Data Review, Demographic Feature Operationalization. Table B in S1 Text—All of US Research Program Detailed Demographics. Box D in S1 Text—Data Types used for SDoH Feature Operationalization. (DOCX) [file pmen.0000015.s001.docx]

The Role of Social Determinants of Health and Social Position in Mental Health: An Examination of the Moderating Effects of Race, Ethnicity, and Gender on Depression through the All of Us Dataset

Supporting Information

Contents

[Box A in S1 Text - Connections between SDoH and Depression 1](#_Toc171643001)

[Box B in S1 Text - All of Us Program Research Program Measurements 4](#_Toc171643002)

[Table A in S1 Text - All of Us Research Program Survey Design 6](#_Toc171643003)

[Box C in S1 Text - AoU Research Program Data Review, Demographic Feature Operationalization 12](#_Toc171643004)

[Table B in S1 Text - All of US Research Program Detailed Demographics 14](#_Toc171643005)

[Box D in S1 Text - Data Types used for SDoH Feature Operationalization 17](#_Toc171643006)

# Box A in S1 Text - Connections between SDoH and Depression

This supplement provides additional details about the connections between food insecurity, neighborhood social cohesion, discrimination, and loneliness on depression that are the focus of the current study.

**Food Insecurity**. A study focused on food insecurity and its connection to depression examined 131 college students aged 18 to 24 in Mississippi [1]. The researchers employed the US Department of Agriculture's Household Food Security Survey Module: Six-Item Short Form to assess food security and used the Patient Health Questionnaire-9 to measure depression. The findings revealed that African American students were more likely to experience food insecurity compared to their Caucasian peers. Moreover, students facing severe food insecurity had a 4.52-times higher likelihood of developing depression compared to those with more food security.

**Neighborhood Social Cohesion**. Another study delved into the relationship between neighborhood cohesion and psychological distress, focusing on various racial/ethnic and sexual orientation groups [2]. The researchers defined neighborhood cohesion as the feeling of connectedness to one's neighborhood, which fosters supportive relationships. The results showed that neighborhood cohesion had a more significant protective effect for heterosexual groups, particularly against moderate psychological distress, in comparison to their non-heterosexual counterparts. This was evident in differences like an 8.16 predicted probability gap for moderate distress between white non-LGB individuals and LGB white individuals. Additionally, neighborhood cohesion seemed to be a protective factor against severe psychological distress, especially for lesbian, gay, and bisexual groups.

**Discrimination**. Swann et al. (2020) conducted a study focusing on enacted stigma based on race/ethnicity and sexual/gender minority (SGM) status as predictors of mental health outcomes and alcohol-related problems [3]. Their evaluation encompassed the effects of racial discrimination, SGM victimization, and sexual orientation microaggressions on various symptoms, including depression and anxiety. In their sample, they found that 45.1% of participants reported no recent victimization experiences, while only 2.3% said the same about microaggressions. This suggests that subtler stigmatizing behaviors like microaggressions are experienced more frequently by SGM individuals than overt victimization and, therefore, may be just as likely to have a detrimental impact on their mental health.

Multifactorial discrimination has been shown to be a fundamental contributor to mental health disparities in a study utilizing baseline data from Project STRIDE: Stress, Identity, and Mental Health [4]. Multifactorial discrimination encompasses discrimination experienced across multiple identity markers, including sexual orientation, gender, race/ethnicity, and others. The study found that multifactorial discrimination was a significant risk factor for high depression scores, particularly in terms of chronic strain and the total number of stressful life events. Notably, women and Latino/Hispanic individuals were more likely to have high depression scores, highlighting the involvement of social identities and their influence on mental health. Additionally, individuals with low depression scores still experienced impacts on anxiety and aggregate mental health scores due to discrimination.

**Loneliness**. Significant research has examined the strong connection between loneliness and depression examining the role in both young adults [5] and the elderly [6]. Indeed, loneliness and depression are often comorbid and connected with other factors like education, wealth, and status [7]. Jeste and Pender (2022) note, “homelessness, social isolation with associated loneliness, social media, positive childhood experiences, social connections, and community-level resilience” as factors that exacerbate outcomes for patients with existing serious mental illnesses and substance use disorders [8]. It may be noted that the elderly are significantly more vulnerable to loneliness and social isolation than the rest of the population and therefore may be at greater risk for depression induced by loneliness [6]. Their lack of social connectedness, at least in part, may be attributed to loss of life of partners and friends, decreased physical mobility, and chronic conditions [9,10].

1. Reeder N, Tapanee P, Persell A, Tolar-Peterson T. Food Insecurity, Depression, and Race: Correlations Observed Among College Students at a University in the Southeastern United States. Int J Environ Res Public Health. 2020;17: 8268. doi:10.3390/ijerph17218268

2. Miller GH, Marquez-Velarde G, Lindstrom E-D, Keith VM, Brown LE. Neighborhood cohesion and psychological distress across race and sexual orientation. SSM - Popul Health. 2022;18: 101134. doi:10.1016/j.ssmph.2022.101134

3. Swann G, Stephens J, Newcomb ME, Whitton SW. Effects of sexual/gender minority- and race-based enacted stigma on mental health and substance use in female assigned at birth sexual minority youth. Cultur Divers Ethnic Minor Psychol. 2020;26: 239–249. doi:10.1037/cdp0000292

4. Khan M, Ilcisin M, Saxton K. Multifactorial discrimination as a fundamental cause of mental health inequities. Int J Equity Health. 2017;16: 43. doi:10.1186/s12939-017-0532-z

5. Achterbergh L, Pitman A, Birken M, Pearce E, Sno H, Johnson S. The experience of loneliness among young people with depression: a qualitative meta-synthesis of the literature. BMC Psychiatry. 2020;20: 415. doi:10.1186/s12888-020-02818-3

6. Isik K, Başoğul C, Yildirim H. The relationship between perceived loneliness and depression in the elderly and influencing factors. Perspect Psychiatr Care. 2021;57: 351–357. doi:10.1111/ppc.12572

7. Green MJ, Whitley E, Niedzwiedz CL, Shaw RJ, Katikireddi SV. Social contact and inequalities in depressive symptoms and loneliness among older adults: A mediation analysis of the English Longitudinal Study of Ageing. SSM - Popul Health. 2021;13: 100726. doi:10.1016/j.ssmph.2021.100726

8. Jeste DV, Pender VB. Social Determinants of Mental Health: Recommendations for Research, Training, Practice, and Policy. JAMA Psychiatry. 2022;79: 283–284. doi:10.1001/jamapsychiatry.2021.4385

9. Smith ML, Racoosin J, Wilkerson R, Ivey RM, Hawkley L, Holt-Lunstad J, et al. Societal- and community-level strategies to improve social connectedness among older adults. Front Public Health. 2023;11: 1176895. doi:10.3389/fpubh.2023.1176895

10. The Epidemiology of Social Isolation: National Health and Aging Trends Study - PubMed. [cited 17 Jan 2024]. Available: https://pubmed.ncbi.nlm.nih.gov/29590462/

# Box B in S1 Text - All of Us Program Research Program Measurements

The All of Us SDoH survey draws from several instruments.

- Accountable Health Communities Health-Related Social Needs Screening Tool
- Behavioral Risk Factor Surveillance System (BRFSS)
- Brief Multidimensional Measure of Religiousness/Spirituality – Daily Spiritual Experiences Scale Short Form
- California Health Interview Survey
- Cohen’s Perceived Stress Scale
- Discrimination in Medical Settings
- Health Begins Upstream Risk Screening Tool
- National Health and Nutrition Examination Survey (NHANES)
- National Health Interview Survey (NHIS)
- PANES International Prevalence Study (IPS) on Physical Activity
- RAND MOS Social Support Survey Instrument
- Ross-Mirowsky Perceived Neighborhood Disorder Scale
- Social Cohesion Neighborhood Scale
- The Everyday Discrimination Scale
- The Hunger Vital Sign
- UCLA Loneliness Scale
- UK Biobank.

The All of Us Social Determinants of Health Survey questions were aggregated from these validated instruments designed to measure specific social determinants of health. The items “health care access and coverage” are not included in the All of Us Social Determinants of Health survey. These items are covered in The Basics and Health Care Access and Utilization surveys, which were administered before the Social Determinants of Health Survey. We combine selected health care questions from these other surveys with the Social Determinants of Health survey to form the corpus of questions for our analysis of social determinants of health. Listed below in Table S1 are the modules and the questions utilized in the current study.

# Table A in S1 Text - All of Us Research Program Survey Design

| **Module/Survey/Question** | **# Questions** |
| --- | --- |
| **Social Determinants of Health** | **81** |
| **Accountable Health Communities Health-Related Social Needs Screening Tool** | **1** |
| 1. Think about the place you live. Do you have problems with any of the following? Select all that apply. |  |
| **Brief Multidimensional Measure of Religiousness/Spirituality – Daily Spiritual Experiences Scale Short Form** | **7** |
| 1. How often do you desire to be closer to or in union with God (or a higher power)? |  |
| 1. How often do you feel deep inner peace or harmony? |  |
| 1. How often do you feel God's (or a higher power's) love for you directly or through others? |  |
| 1. How often do you feel God's (or a higher power's) presence? |  |
| 1. How often do you feel that you are spiritually touched by the beauty of creation? |  |
| 1. How often do you find strength and comfort in your religion? |  |
| 1. How often do you go to religious meetings or services? |  |
| **California Health Interview Survey** | **2** |
| 1. Do you speak a language other than English at home? |  |
| 1. Since you speak a language other than English at home, we are interested in your own thoughts about how well you think you speak English. Would you say you speak English... |  |
| **Cohen’s Perceived Stress Scale** | **10** |
| 1. In the last month how often have you been able to control irritations in your life? |  |
| 1. In the last month how often have you been angered because of things that were outside of your control? |  |
| 1. In the last month how often have you been upset because of something that happened unexpectedly? |  |
| 1. In the last month how often have you felt confident about your ability to handle your personal problems? |  |
| 1. In the last month how often have you felt difficulties were piling up so high that you could not overcome them? |  |
| 1. In the last month how often have you felt nervous and "stressed"? |  |
| 1. In the last month how often have you felt that things were going your way? |  |
| 1. In the last month how often have you felt that you were on top of things? |  |
| 1. In the last month how often have you felt that you were unable to control the important things in your life? |  |
| 1. In the last month how often have you found that you could not cope with all the things that you had to do? |  |
| **Discrimination in Medical Settings** | **7** |
| 1. How often are you treated with less courtesy than other people when you go to a doctor's office or other health care provider? |  |
| 1. How often are you treated with less respect than other people when you go to a doctor's office or other health care provider? |  |
| 1. How often do you feel like a doctor or nurse is not listening to what you were saying. when you go to a doctor's office or other health care provider? |  |
| 1. How often do you receive poorer service than others when you go to a doctor's office or other health care provider? |  |
| 1. How often does a doctor or nurse act as if he or she is afraid of you when you go to a doctor's office or other health care provider? |  |
| 1. How often does a doctor or nurse act as if he or she is better than you when you go to a doctor's office or other health care provider? |  |
| 1. How often does a doctor or nurse act as if he or she thinks you are not smart when you go to a doctor's office or other health care provider? |  |
| **Health Begins Upstream Risk Screening Tool** |  |
| 1. In the last 12 months how many times have you or your family moved from one home to another? Number of moves in past 12 months: |  |
| **PANES International Prevalence Study (IPS) on Physical Activity** | **8** |
| 1. It is within a 10–15-minute walk to a transit stop (such as bus train trolley or tram) from my home. Would you say that you... |  |
| 1. Many shops stores markets or other places to buy things I need are within easy walking distance of my home. Would you say that you... |  |
| 1. My neighborhood has several free or low-cost recreation facilities such as parks walking trails bike paths recreation centers playgrounds public swimming pools etc. Would you say that you... |  |
| 1. The crime rate in my neighborhood makes it unsafe to go on walks at night. Would you say that you... |  |
| 1. The crime rate in my neighborhood makes it unsafe to go on walks during the day. Would you say that you... |  |
| 1. There are facilities to bicycle in or near my neighborhood such as special lanes separate paths or trails or shared use paths for cycles and pedestrians. Would you say that you... |  |
| 1. There are sidewalks on most of the streets in my neighborhood. Would you say that you... |  |
| 1. What is the main type of housing in your neighborhood? |  |
| **RAND MOS Social Support Survey Instrument** | **9** |
| 1. How much you agree or disagree that people around here are willing to help their neighbor? |  |
| 1. How often do you have someone to have a good time with? |  |
| 1. How often do you have someone to help you if you were confined to bed? |  |
| 1. How often do you have someone to help you with daily chores if you were sick? |  |
| 1. How often do you have someone to love and make you feel wanted? |  |
| 1. How often do you have someone to prepare your meals if you were unable to do it yourself? |  |
| 1. How often do you have someone to take you to the doctor if you need it? |  |
| 1. How often do you have someone to turn to for suggestions about how to deal with a personal problem? |  |
| 1. How often do you have someone who understands your problems? |  |
| **Ross-Mirowsky Perceived Neighborhood Disorder Scale** | **13** |
| 1. How much you agree or disagree that in your neighborhood people watch out for each other? |  |
| 1. How much you agree or disagree that people in your neighborhood take good care of their houses and apartments? |  |
| 1. How much you agree or disagree that there are lot of abandoned buildings in your neighborhood? |  |
| 1. How much you agree or disagree that there are too many people hanging around on the streets near your home? |  |
| 1. How much you agree or disagree that there is a lot of crime in your neighborhood? |  |
| 1. How much you agree or disagree that there is a lot of graffiti in your neighborhood? |  |
| 1. How much you agree or disagree that there is too much alcohol use in your neighborhood? |  |
| 1. How much you agree or disagree that there is too much drug use in your neighborhood? |  |
| 1. How much you agree or disagree that vandalism is common in your neighborhood? |  |
| 1. How much you agree or disagree that you are always having trouble with your neighbors? |  |
| 1. How much you agree or disagree that your neighborhood is clean? |  |
| 1. How much you agree or disagree that your neighborhood is noisy? |  |
| 1. How much you agree or disagree that your neighborhood is safe? |  |
| **Social Cohesion Neighborhood Scale** | **3** |
| 1. How much you agree or disagree that people in your neighborhood can be trusted? |  |
| 1. How much you agree or disagree that people in your neighborhood generally get along with each other? |  |
| 1. How much you agree or disagree that people in your neighborhood share the same values? |  |
| **The Everyday Discrimination Scale** | **10** |
| 1. Discrimination: What do you think is the main reason for these experiences? |  |
| 1. In your day-to-day life how often are you called names or insulted? |  |
| 1. In your day-to-day life how often are you threatened or harassed? |  |
| 1. In your day-to-day life how often are you treated with less courtesy than other people? |  |
| 1. In your day-to-day life how often are you treated with less respect than other people? |  |
| 1. In your day-to-day life how often do people act as if they are afraid of you? |  |
| 1. In your day-to-day life how often do people act as if they think you are dishonest? |  |
| 1. In your day-to-day life how often do people act as if they think you are not smart? |  |
| 1. In your day-to-day life how often do people act as if they're better than you are? |  |
| 1. In your day-to-day life how often do you receive poorer service than other people at restaurants or stores? |  |
| **The Hunger Vital Sign** | **2** |
| 1. Within the past 12 months were you worried whether the food you had bought just didn't last and you didn't have money to get more? |  |
| 1. Within the past 12 months were you worried whether your food would run out before you got money to buy more? |  |
| **UCLA Loneliness Scale** | **8** |
| 1. How often do you feel isolated from others? |  |
| 1. How often do you feel lack companionship? |  |
| 1. How often do you feel left out? |  |
| 1. How often do you feel that people are around you but not with you? |  |
| 1. How often do you feel that there is no one you can turn to? |  |
| 1. How often do you feel that you are an outgoing person? |  |
| 1. How often do you feel that you are unhappy being so withdrawn? |  |
| 1. How often do you fell that you can find companionship when you want it? |  |
| **The Basics** | **8** |
| **Behavioral Risk Factor Surveillance System (BRFSS)** | **5** |
| 1. Current Home Ownership |  |
| 1. Current Marital Status |  |
| 1. Income: Annual Income |  |
| 1. Living Situation: How Many People |  |
| 1. Living Situation: People Under 18 |  |
| **National Health and Nutrition Examination Survey (NHANES)** | **1** |
| 1. Insurance: Health Insurance |  |
| **UK Biobank** | **2** |
| 1. Living Situation: How many living years |  |
| 1. Living Situation: Stable House Concern |  |
| **Health Care Access and Utilization** | **15** |
| **National Health Interview Survey (NHIS)** | **15** |
| 1. Can't Afford Care: Alternative Therapies |  |
| 1. Can't Afford Care: Bought Rx from Other Country |  |
| 1. Can't Afford Care: Delayed Filling Rx to Save Money |  |
| 1. Can't Afford Care: Dental Care |  |
| 1. Can't Afford Care: Emergency Care |  |
| 1. Can't Afford Care: Follow-up Care |  |
| 1. Can't Afford Care: Healthcare Provider |  |
| 1. Can't Afford Care: Mental Health Counseling |  |
| 1. Can't Afford Care: Prescription Medicines |  |
| 1. Can't Afford Care: Specialist |  |
| 1. Can't Afford Care: Took Less Med to Save Money |  |
| 1. Can't Afford Care: Worried About Paying |  |
| 1. Health Advice: Spoken to Professional |  |
| 1. Insurance: Healthcare Coverage |  |
| 1. Insurance: Insurance Accepted |  |
| **Grand Total** | **104** |

# Box C in S1 Text - AoU Research Program Data Review, Demographic Feature Operationalization

All demographic features were sourced from The Basics survey, which all participants are required to complete. Six factors were selected for our analysis: gender/sexual identity, race/ethnicity, household income, age, education level, and home ownership. These categories are further decomposed into the following features:

1. Gender/sexuality: cisgender heterosexual male, cisgender heterosexual female, LGBTQIA2+

This feature category is a composite of the gender and sexual orientation questions in The Basics survey. Participants were allowed to choose multiple options for the sexual orientation question. LGBTQIA2+ includes those who identified as one or more of the following: bisexual sexual orientation, gay sexual orientation, lesbian sexual orientation, no sexual orientation, nonbinary gender, transgender, or additional options for gender. Cisgender heterosexual male includes only those who identified as straight and male, and cisgender heterosexual female includes only those who identified as straight and female.

1. Race/ethnicity: White, Hispanic/Latinx, Black or African American, Asian, more than one population, or other.

In The Basics survey, All of Us participants were asked to select which racial-ethnic categories describe them. Participants were allowed to select multiple answers to this question. Participants who selected more than one answer are categorized as more than one population. In our analytical sample, Middle Eastern/North African and Native Hawaiian/other Pacific Islander responses are combined to form the “Other” race/ethnicity category.

1. Age: continuous (units = Years)

There is no age field natively encoded in All of Us. However, there are fields for date of birth and time at which a survey was taken. For our analytical sample, we consider the age at which a participant took the Social Determinants of Health survey, in years. Individuals younger than 18 years are not permitted to participate in All of Us.

1. Income: continuous (units = $10,000)

Answers for the household income question were binned into 9 groups: less than $10,000; $10,000-$24,999; $25,000-$34,999; $35,000-49,999; $50,000-$74,999; $75,000-$99,999; $100,000-$149,999; $150,000-$199,999; $200,000 or more. We include this feature as a continuous variable with the value set at the lower end of the interval.

1. Education: college degree, no college degree

Answers for the highest grade of education completed are binned into 8 groups: never attended, grades 1-4, grades 5-8, grades 9-11, grade 12 or GED, one to three years of college, college graduate, advanced degree. We binary encode this feature into college graduate and not a college graduate labels. 66.8% of those in our analytical sample reported college graduate status.

1. Home ownership: homeowner, not a homeowner

The All of Us survey question regarding living arrangement offers three response choices: ownership, rental, or other arrangement. We aggregate the rent and other arrangement labels to create just two labels: homeowner and not a homeowner.

# Table B in S1 Text - All of US Research Program Detailed Demographics

|  |  | AoU Full Dataset | | AoU SDOH Sample | | AoU Analytical Sample | |
| --- | --- | --- | --- | --- | --- | --- | --- |
|  | All Participants | 413360 |  | 117780 |  | 86960 |  |
| Gender/Sexual Identity | Cisgender Heterosexual Male | 136340 | 33% | 35340 | 30% | 27580 | 32% |
|  | Cisgender Heterosexual Female | 220420 | 53% | 65960 | 56% | 50060 | 58% |
|  | LGBTQAI2+ | 37860 | 9% | 11600 | 10% | 9320 | 11% |
|  | Missing | 18720 | 5% | 4880 | 4% | 0 | 0% |
| Race/Ethnicity | White | 222640 | 54% | 87880 | 75% | 69340 | 80% |
|  | Hispanic/Latinx | 64680 | 16% | 7980 | 7% | 4920 | 6% |
|  | Black or African American | 77060 | 19% | 8980 | 8% | 5980 | 7% |
|  | Asian | 13840 | 3% | 3120 | 3% | 2340 | 3% |
|  | Middle Eastern or North African | 2360 | 1% | 460 | <1% | 340 | <1% |
|  | Native Hawaiian or Other Pacific Islander | 420 | <1% | 60 | <1% | 40 | <1% |
|  | More than One Population | 16120 | 4% | 4220 | 4% | 3260 | 4% |
|  | None of These | 4380 | 1% | 1040 | 1% | 740 | 1% |
|  | Missing | 11700 | 3% | 4040 | 3% | 0 | 0% |
| Education: Highest Grade | Never Attended | 560 | <1% | ≤20 | <1% | ≤20 | <1% |
|  | 1-4 | 3320 | 1% | 220 | <1% | 80 | <1% |
|  | 5-8 | 8780 | 2% | 720 | 1% | 320 | <1% |
|  | 9-11 | 23760 | 6% | 1640 | 1% | 920 | 1% |
|  | 12 or GED | 77020 | 19% | 10700 | 9% | 7040 | 8% |
|  | College One to Three Years | 104140 | 25% | 27440 | 23% | 20520 | 24% |
|  | College Graduate | 93240 | 23% | 33920 | 29% | 26680 | 31% |
|  | College Advanced Degree | 89100 | 22% | 39700 | 34% | 31420 | 36% |
|  | Missing | 13440 | 3% | 3460 | 3% | 0 | 0% |
| Age | 18-29 | 41240 | 9% | 7460 | 6% | 5320 | 6% |
|  | 30-39 | 65140 | 15% | 13000 | 11% | 10260 | 12% |
|  | 40-49 | 63780 | 14% | 14080 | 12% | 10940 | 13% |
|  | 50-59 | 80500 | 18% | 19000 | 16% | 14520 | 17% |
|  | 60-69 | 96400 | 22% | 28840 | 25% | 21320 | 25% |
|  | 70-79 | 71120 | 16% | 27780 | 24% | 19800 | 23% |
|  | 80-89 | 21320 | 5% | 6980 | 6% | 4440 | 5% |
|  | 90+ | 2720 | 1% | 620 | 1% | 340 | <1% |
| Household Income | less than $10,000 | 55540 | 13% | 5440 | 5% | 4060 | 5% |
|  | $10,000 - $24,999 | 47180 | 11% | 9420 | 8% | 7380 | 9% |
|  | $25,000 - $34,999 | 29280 | 7% | 7540 | 6% | 6080 | 7% |
|  | $35,000 - $49,999 | 32540 | 8% | 10280 | 9% | 8520 | 10% |
|  | $50,000 - $74,999 | 43600 | 11% | 16640 | 14% | 14020 | 16% |
|  | $75,000 - $99,999 | 33900 | 8% | 14320 | 12% | 12220 | 14% |
|  | $100,000 - $149,999 | 41780 | 10% | 18660 | 16% | 16180 | 19% |
|  | $150,000 - $199,999 | 19660 | 5% | 9000 | 8% | 7780 | 9% |
|  | $200,000 or more | 26880 | 7% | 12220 | 10% | 10740 | 12% |
|  | Missing | 83020 | 20% | 14260 | 12% | 0 | 0% |
| Home Ownership | Own Home | 189580 | 46% | 77620 | 66% | 60400 | 70% |
|  | Rent | 159840 | 39% | 28980 | 25% | 21480 | 25% |
|  | Other Arrangement | 42480 | 10% | 7100 | 6% | 5080 | 6% |
|  | Missing | 21460 | 5% | 4100 | 4% | 0 | 0% |

# Box D in S1 Text - Data Types used for SDoH Feature Operationalization

The following section provides additional information to supplement Table 2 in the manuscript about the data types were used in the current study to operationalize SDoH features.

**Data Type 1: Mean Subscale**

The mean subscale encoding reflects the fact that our social determinants of health fields partition survey questions by the unique survey instruments from which they were sourced. Rather than use the response to each individual question in our analysis, we create a single score for each sub-survey, allowing us to represent each social determinant of health field with a single value. This is performed as follows:

1. Likert scale responses are encoded as integer values 1 through *n*, where *n* is the number of possible responses on the scale. For example, for the question asking, “How much you agree or disagree that your neighborhood is clean?” the responses strongly disagree, disagree, agree, and strongly agree are mapped to 1, 2, 3, and 4 respectively.
2. Instances where participants skipped, preferred not to answer, or otherwise did not answer a question are recorded as null values in the dataset.
3. Questions within each field originate from a consistent source and were thematically aligned. Generally, the questions in a given field share a similar valence where higher scale values represent positive outcomes and lower values denote negative outcomes. In instances where answer scales diverge in valence, the values for certain questions are inverted to ensure uniformity in the direction of encoding across all questions within the field.
4. All scaled responses are re-scaled using a min-max standard scaler so that their values fall between 0 and 1.
5. Each field contains some number of questions, but some participants did not respond to all the questions in a given field. If a field contains three or fewer questions, we require participants to respond to all questions in the field to be included in the analytical sample. If a field contains more than three questions, we require participants to respond to at least three questions to be included in the analytical sample. We set this threshold to allow participants who did not answer all of the questions in a given field to remain in the analytical sample.
6. We let each field equal the mean of the responses to its questions. Because question responses are re-scaled to fall between 0 and 1, each field is equal to a value between 0 and 1. Null values are omitted from the mean value calculation.

**Data Type 2: Numeric Response**

The Transience field is encoded as a numeric response from a single question: “In the last 12 months how many times have you or your family moved from one home to another?” We simply take the response to this question as the field value.

**Data Type 3: Sum of Checked Responses**

Two fields are encoded as sums of checked responses: Housing Issues and Lack of Health Care Access. These fields reflect the response to one question each. These questions accept “check all that apply” responses. We take the sum of the number of checked responses as the field value. For example, the Housing Issues field uses the question from the Social Determinants of Health Survey, “Think about the place you live. Do you have problems with any of the following?” Excluding the “None of the above response,” we sum the number of checked responses, indicating the number of relevant problems in the home, and take that as the field value. These values are then re-scaled by the min-max scaler.

**Data Type 4: Indicator**

Only the Health Insurance field takes an indicator value. In this field, 1 represents an affirmative response to the question, “Are you covered by health insurance or some other kind of health care plan?” and 0 represents otherwise.

After removing those participants who did not take the Social Determinants of Health survey or had null values in their demographic or SDoH fields, we are left with an analytical sample of size 86,960.
